# Stakeholders’ perceptions and experiences of the National Health Service diabetes prevention programme in England: qualitative study with service users, intervention providers and deliverers, commissioners and referrers

**DOI:** 10.1186/s12913-020-05160-2

**Published:** 2020-04-15

**Authors:** Angela M. Rodrigues, Anna Haste, Linda Penn, Ruth Bell, Carolyn Summerbell, Martin White, Ashley J. Adamson, Falko F. Sniehotta

**Affiliations:** 1 Department of Psychology, Northumbria University, Northumberland Building, Newcastle upon Tyne, NE1 8ST UK; 2 Population Health Sciences Institute, Newcastle University, Baddiley Clark Building, Newcastle upon Tyne, NE2 4AX UK; 3 Fuse: UKCRC Centre for Translational Research in Public Health, Newcastle upon Tyne, UK; 4 Department of Psychology, School of Social Sciences, Humanities and Law, Teesside University, Middlesbrough, TS1 3BX UK; 5 Human Nutrition Research Centre, Newcastle University, Newcastle upon Tyne, UK; 6 Faculty of Social Sciences and Health, Durham University, Durham City, DH1 3HN UK; 7 MRC Epidemiology Unit, University of Cambridge, Cambridge Biomedical Campus, Cambridge, CB2 0QQ UK; 8 NIHR Policy Research Unit Behavioural Science, Baddiley Clark Building, Newcastle upon Tyne, NE2 4AX UK

**Keywords:** Type 2 diabetes, Health policy, National diabetes prevention programme, Public health

## Abstract

**Background:**

The National Health Service diabetes prevention programme in England, (NHS DPP) aims to identify people at high risk of type 2 diabetes (T2D) and offer them a face-to-face, group-based, behaviour change intervention for at least 9 months. The NHS DPP was rolled out in phases. We aimed to elicit stakeholders’ perceptions and experiences of the factors influencing implementation of, and participation in, the programme during the development phase.

**Methods:**

Individual, semi-structured telephone interviews were conducted with 50 purposively sampled stakeholders: service users (*n* = 20); programme commissioners (*n* = 7); referrers (*n* = 8); and intervention deliverers (*n* = 15). Topic guides were structured using a pragmatic, theory-informed approach. Analysis employed the framework method.

**Results:**

We identified factors that influenced participation: *Risk communication at referral -* stakeholders identified point of referral as a window of opportunity to offer brief advice, to provide an understanding of T2D risk and information about the programme; *Perceived impact of the NHS DPP* - service users highlighted the positive perceived impact on their behaviour change, the peer support provided by participating in the programme, the option to involve a relative, and the ‘knock on’ effect on others. Service users also voiced disappointment when blood test results still identified them at high risk after the programme; and *Behavioural maintenance* - participants highlighted the challenges linked to behavioural maintenance (e.g. discontinuation of active support). Factors influencing implementations were also identified: *Case finding* – stakeholders suggested that using community involvement to identify service users could increase reach and ensure that the workload was not solely on GP practices; *Adaptability*: intervention deliverers acknowledged the need to tailor advice to service users’ preferences and needs; *Accountability* – the need to acknowledge who was responsible for what at different stages of the NHS DPP pathway; and *Fidelity* – stakeholders described procedures involved in monitoring service users’ satisfaction, outcome data collection and quality assurance assessments.

**Conclusions:**

The NHS DPP offers an evidence-informed behavioural intervention for T2D prevention. Better risk communication specification could ensure consistency at the referral stage and improve participation in the NHS DPP intervention. Cultural adaptations and outreach strategies could ensure the NHS DPP contributes to reducing health inequalities.

## Introduction

Diabetes affects 6 % of the UK population, with type 2 diabetes (T2D) accounting for approximately 90 % of cases [1]. Diabetes is a leading cause of morbidity and mortality in the UK and costs the NHS £ 8.8 billion a year [2].

Risk of developing T2D is associated with obesity and physical inactivity [3, 4]. A review and meta-analysis of randomised trials evidence showed a pooled effect hazard ratios for T2D incidence in intervention trials with diet and exercise combined of 0.49 (95% CI 0.36 to 0.65) in adults with impaired glucose tolerance [5]. The first T2D prevention RCT in England was based on the Finnish Diabetes Prevention Study (DPS) protocol and demonstrated a similar T2D risk reduction of 55% in the intervention compared with the control group [6]. Despite evidence of effectiveness from randomised controlled trials (RCT), diabetes prevention interventions have not previously been systematically implemented in practice. Therefore, there is a need to identify the challenges of translating the evidence on prevention strategies into feasible programmes [7].

The NHS Diabetes Prevention Programme (NHS DPP) was proposed in the NHS Five Year Forward View [8] and is being led by a partnership between the NHS, Public Health England (PHE) and Diabetes UK (D-UK). The NHS DPP is a national programme that aims to prevent the onset of T2D in high risk individuals and thus reduce their risk of diabetes related complications. The NHS DPP is informed by research evidence and National Institute for Health and Care Excellence (NICE) guidance (PH38) [4, 9].

The NHS DPP intervention was designed to focus on diabetes prevention, and to be informed by proven DPP models, such as the US DPP [10, 11] and the Finnish DPP [12, 13]. PHE also commissioned evidence reviews to underpin the development of the NHS DPP intervention content and delivery [14]. One of these reviews [15] identified specific components which are associated with increased effectiveness in interventions to promote change in diet and/or physical activity. Specifically, the review recommended the inclusion of a set of behaviour change techniques (e.g. motivational interviewing, self-regulatory techniques, prompting self-talk); engaging support from a family member, friend or carer; targeting both diet and physical activity; maximising intensity (number or frequency of contacts); and the use of self-monitoring alongside other self-regulatory techniques (goal-setting, providing feedback, review goals, relapse prevention).

Taken together, this evidence has been translated into the ‘real-world’ NHS DPP by offering ongoing tailored advice, support and encouragement to help people: 1) undertake a minimum of 150 min of ‘moderate-intensity’ physical activity per week; 2) gradually lose weight to reach and maintain a BMI within the healthy range; 3) increase their consumption of wholegrains, vegetables and other foods that are high in dietary fibre; and 4) reduce the total amount of fat in their diet. Details of the intervention programme as provided by the NHS DPP service specification can be found in Table 1 (TIDieR table).

**Table 1:** TIDieR table [16] for the NHS DPP intervention specification in demonstrator sites

TIDieR checklist item	NHS DPP intervention specification
**What**	The primary focus of the NHS DPP is to prevent Type 2 diabetes and aimed at achieving three core goals: • Weight loss, or the maintenance of a healthy weight; • Achievement of UK dietary recommendations related to fibre, fruit and vegetables, oily fish, saturated fat, salt and free sugars; and • Achievement of the England Chief Medical Officer’s (CMO) physical activity recommendations. Goal setting and self-regulation techniques can be used to support users. Family or peer support is accommodated where this would be helpful to a service user
**Who delivered**	Health professionals or other suitably trained and competent individuals.
**How**	Group sessions, with a maximum of 20 people in each group, delivered face-to-face. Individual sessions (either in person or remotely) may also be included to enhance delivery. Alternative approach (e.g., online delivery) is also possible for remote communities that are unable to attend regular face-to-face sessions.
**Where**	Sessions offered at a range of times, days and venues in order to maximise access.
**When and How much**	A minimum of 9 months’ duration; At least 13 sessions, spread across a minimum total of 16 h’ contact time; Each session must last between 1 and 2 h. Follow up every 3 months for 2 years.
**Tailoring**	Goals tailored to suit individual service user requirements for physical activity, weight management and dietary changes.
**Fidelity**	Fidelity to core components should be reported using quality assurance frameworks embedded within data systems and implementation of the intervention. Systems should monitor and maintain the quality through routine checks on intervention delivery.

During the development phase, seven NHS DPP demonstrator sites (Birmingham, Bradford, Durham, Herefordshire, Medway, Salford, and Southwark) were commissioned to provide a variety of T2D prevention programme models in different populations, offering examples of intervention service delivery to inform subsequent national implementation. The selected demonstrator sites were expected to implement a number of processes and strategies, detailed in the draft NHS DPP service specification, in order to test their feasibility and acceptability in practice. These included: identification of eligible participants, risk assessment, risk communication and recruitment of eligible participants, delivery of behavioural interventions, post intervention assessment, data-collection and integration with general practice (Details about demonstrator site interventions can be found in Additional file 1).

Understanding the pragmatic application of effective diabetes prevention programmes is crucial as implementation strategies often experience practical problems [17]. A systematic review of 38 studies with the goal of identifying factors leading to successful implementation of DPP in “real-world” settings [18] suggested that program planners and implementers should aim to design high-intensity program with frequent contacts, if the primary target is weight loss; and/or lower frequency of contacts but with a program duration of at least 12 months, if the primary aim is diabetes risk reduction.

Problems with low uptake and participation are common in diabetes prevention programmes [19–21]. Therefore, greater understanding of people’s views of intervention delivered in the ‘real world’ is needed.

With this in mind, our evaluation of the demonstrator site phase of the NHS DPP aimed to investigate current procedures and inform the national implementation. It involved a number of strands of research: with the overview paper reported elsewhere [22]. Evaluation and refinement of the demonstrator phase of the NHS DPP has been used to improve the quality and effectiveness of the programme. This paper reports findings from qualitative interviews with NHS DPP stakeholders. We aimed to elicit stakeholders’ perceptions and experiences of participating in the intervention and implementing the NHS DPP.

## Methods

### Study design

We undertook a qualitative investigation using theoretically informed one-to-one telephone interviews. We aimed to explore issues related to participation and implementation of the NHS DPP. We used the theoretical domains framework (TDF) [23] to inform our initial approach in developing the topic guides (Additional file 2) in line with previous work in this area [24], and to ensure a broad and comprehensive list of potential influences. The TDF has been useful in previous research looking at implementation processes [25]. This is a specific approach designed to identify relevant theoretical domains that can be perceived as barriers or facilitators to behaviour change. The TDF provides a broad and comprehensive summary of the key behavioural science explanations for behaviour around ‘knowledge’, ‘beliefs about consequences’, ‘beliefs about capabilities’, ‘skills’, ‘environmental context & resources’, ‘social influences’, ‘memory, attention & decision processes’, ‘behavioural regulation’, ‘emotion’, ‘social or professional role/identity’, ‘motivation & goals’ and the nature of the behaviour [23].

We used the Standards for Reporting Qualitative Research (SRQR) checklist from the EQUATOR Network website as the reporting guideline for this qualitative study.

### Participants

The NHS DPP management group identified a key contact person at each of seven demonstrator sites, who helped to identify potential interviewees from all stakeholder groups. After obtaining permission, eligible participants were approached individually (via email) by researchers and informed about the study, using an opt-in procedure. They were also provided with a participant information sheet explaining the purpose of the study. In total the following stakeholders provided contact details and were approached by the research team: 25 service users, 17 deliverers, 19 commissioners and 18 referrers.

Purposive sampling was used to achieve maximum variation among participants with respect to professional grouping, age and gender as appropriate. Commissioners and deliverers interviewed represented each of the seven sites. Referrers interviewed represented four of the demonstrator sites. The sampling strategy for service users ensured variation with respect to geographical location, age and gender. The majority of service users were interviewed nearer the end of the invention or after completion. Service users interviewed represented four of the demonstrator sites.

Consent forms were received electronically from all interviewees prior to the interview. If this was not possible audio recording of consent was obtained and recorded separately from the interview audio recording.

A total of fifty interviews were conducted, drawing participants from four stakeholder groups:

A. NHS DPP service users [adults at high-risk of T2D defined as having NDH (HbA1c 42–47 mmol/mol (6.0–6.4%) or FPG 5.5–6.9 mmol/mol)] (*n* = 20, 80% interview completion rate);
B. NHS DPP intervention providers and deliverers (i.e. primary care and local authority staff, volunteer health champions, health trainers, and fitness trainers) (*n* = 15, 88% interview completion rate);
C. Local authority, public health and CCG programme commissioners (*n* = 7, 37% interview completion rate);
D. Those referring participants to NHS DPP, who included: general practitioners, nurses, practice managers and health care assistants (*n* = 8, 44% interview completion rate).

### Data collection

One-to-one semi-structured interviews were conducted via telephone between February and June 2016. Due to the various locations involved in this project, this design enabled the geographical flexibility needed to interview participants for all 7 sites at their convenience. All interviews were conducted by three female researchers (AR, AH, LP) with experience in interviewing and without previous relationship with research participants.

Topic guides were followed and iteratively developed in response to feedback from early participant interactions. Interviews lasted between 15 and 60 min. All interviews were digitally audio recorded and transcribed verbatim for analysis. Transcripts were checked and anonymised by the research team. Interviews were continued until data saturation was achieved [26].

### Data analysis

Our data analysis approach used inductive thematic analysis that allowed themes to emerge from the data, an approach regularly used in TDF interviews [27]. Given that the topic guides were based on the TDF, some emerging themes reflected the TDF domains (e.g., maintenance).

Framework method was used to analyse the interview transcripts and to identify themes [28, 29]. Transcripts was read several times by the researchers (AH, AR, LP) and were coded line by line and analysed to identify similarities and differences. Emerging themes were identified relating to facilitators and barriers to the NHS DPP participation and implementation and a thematic framework based on a sample of transcripts was created. This framework was then indexed and mapped across all transcripts, adapting the framework along the way when needed. All transcripts were coded by two researchers (AH, AR) and a subset of interviews were independently coded and analysed by a third researcher (LP). Any divergences were resolved by consensus involving a fourth researcher (FFS). The coding framework was then discussed and agreed within the core NHD DPP evaluation team (AH, AR, LP, FFS). NVivo software was used to facilitate coding and analysis of transcribed data.

The data saturation analysis was conducted in two steps. First, we created a map of all the themes that emerged from the interviews (from an initial sample of three transcripts). Using this framework, we then conducted further interviews until no additional material was retrieved from the final transcripts from all four stakeholder groups.

## Results

### Participants and descriptive data

The majority of participants were white British (88%) and female (62%) (Table 2).

**Table 2:** Characteristics of interview participants

	Service Users	Intervention Providers/ Deliverers	Commissioners	Referrers	Total
**Age:**					
Mean	65.7	45.8	52.5	36.6	53.9
Min-Max	46–77	29–67	33–63	22–61	22–77
**Gender [No (%)]:**					
Male	9 (45)	4 (26.7)	3 (43)	3 (37.5)	19 (38)
Female	11 (55)	11 (73.3)	4 (57)	5 (62.5)	31 (62)
**Ethnicity [No (%)]:**					
White British	18 (90)	12 (80)	7 (100)	7 (87.5)	44 (88)
Other	2 (10)	3 (20)	0 (0)	1 (12.5)	6 (12)

### Main results

Factors associated with participation and implementation of the NHS DPP were identified in relation to enrolment and reach, perceived impact of the NHS DPP on service users, maintenance of behavioural change, case identification procedures, and adaptability of the intervention.

#### Factors influencing participation

##### Risk communication at referral

For service users a main barrier emerged during the period between referral and the first session. This was described as a daunting experience as the programme was ‘the unknown’. When information was provided by various different health professionals it could often confuse users as to who or what they were being referred to.

*“The hard part I think is signing up to it because you don’t know what it’s all about. I was a bit wary because I thought, “is it going to be very grim or very intensive or are they going to make me do all sorts of activities I don’t want to do?” but it wasn’t like that at all...” (Service User 3)*

Service users highlighted the need for a full explanation about the meaning of the diagnosis and the need for the health care professionals to explain this effectively. Service users also spoke about encounters with health care professionals who had informed them that developing T2D was inevitable, instead of informing them about preventative measures.

*“The nurses told me that I probably wouldn’t be able to avoid it (..)” (Service User 3)*

The perceived impact of being told they were at risk was more commonly met with surprise and concern mainly related to a lack of knowledge about diabetes, and also uncertainty concerning what to expect from the diabetes prevention programme.

*“I was horrified actually. I thought, “Me. No they’re speaking about somebody else. This can’t be me because no family have ever had diabetes.” Of course with that I just hadn’t given it a thought and really quite shocked.” (Service User 20)*

Inconsistency in the terminology used when providing users with test results was also highlighted. Deliverers stated that the impaired glucose regulation (IGR) terminology was difficult to understand for some users and could cause confusion.

*“I mean IGR from what I can gather is a new terminology being used on pre-diabetes. It’s definitely been publicised a lot more recently, which I think is fantastic. Previous research, from what I’ve read, it spoke about impaired glucose tolerance, IGT or glucose fasting, IGF. Sometimes the terminology used and I’m not that clear.” (Deliverer 10)*

Deliverers also advised on the importance of raising more awareness in terms of diabetes and its causes since most service users lacked information about the condition. Deliverers also identified users’ reaction to being labelled as ‘high risk’ as a barrier that could lead to lack of motivation to be involved in the programme.

*“I don’t think many people really understand what diabetes is, what the risk factors are, and the fact that they might be at risk, so I think there needs to be some national recognition of that and awareness-raising of it as well.” (Deliverer 1)*

##### Perceived impact of the NHS DPP

The factors included in this section relate to the anticipated benefits that influenced the take up of the NHS DPP, experienced benefits that influenced ongoing participation and perceived benefits resulting from participation in the NHS DPP.

##### Behaviour change

Participants described how the anticipated benefits in terms of behaviour change influenced the take up of the NHS DPP. Participants believed they were fortunate to have had the opportunity, a chance to prove to themselves and others that they could improve their health behaviours. Users also mentioned that being labelled as high risk and enrolling in the programme made them realise how important behaviour change was for diabetes prevention.

*“The only impact was in actually getting me rolling up my sleeves and doing something about something that I really should have been doing for some time.” (Service User 7)*

*“I feel I’ve been extremely lucky to be given the opportunity to have this course. It certainly has worked and made me carry on.” (Service User 20)*

Deliverers described the programme as ‘lifesaving’ for users. Service users described the programme as a window of opportunity to reduce their risk of T2D by changing their lifestyle and improving general wellbeing.

*“The fact that clients are given that window of opportunity to reduce their risk factors, not only for themselves, not only can they benefit from it but the fact that it will save the NHS a lot of money in terms of the cost of managing diabetes.” (Deliverer 2)*

Service users also described factors that influenced their ongoing participation on the programme. Service users felt motivated and confident to maintain the behaviour change and to continue to improve their health.

*“I wouldn't like to think that now I've started, to stop everything (…) I said I would like to keep it going because I found so much improvement in myself. (…) I feel more confident than I did when I started.” (Service User 14)*

Observing users’ lifestyle change journey and the insight gained through the programme about behaviour change (e.g. how to eat healthily) was viewed as important by deliverers.

*“When somebody has a lightbulb moment and you can see it, it is worth everything really. It can just be one silly little thing, you know, from not knowing the different types of sugar names and they go, “Really? Oh I’ve been using honey because I thought it was better than sugar.” Just a little thing like that. But I think that is what it is about actually.” (Deliverer 5)*

##### Social support

Forming lasting social networks was described by service users as an important factor for ongoing participation in the programme. Group support (e.g. other users, deliverers) was highlighted as an essential feature of the NHS DPP sessions. Support from other group members allowed sharing of similar experiences and troubleshooting during the sessions.

*“We’re all in the same boat, yes, it’s lovely. I’ll miss them when it’s finished.” (Service User 6)*

Support from family and friends ranged from awareness that users were on the programme to implementing the lifestyle changes as a couple or family.

*“As far as the diet was concerned at the time my sons were on a diet, everyone was on a diet in the house.” (Service User 16)*

In some demonstrator sites, service users were given the opportunity to invite a significant other to the sessions and this was described as an effective strategy to increase the involvement of specific groups in the programme.

*“They're allowed to bring somebody for support if they wish (…).” (Deliverers 11)*

Support was also gained from the intervention deliverer who provided helpful suggestions, especially when other health conditions were present among service users. Service users also enjoyed the regular contact and monitoring as it kept them on track.

*“I think some of the group work as well from the feedback that we have had that has been very well received. They have built up quite a good rapport and supportive mechanism as well off the back of that” (Commissioner 7)*

##### Interaction with others

The programme provided an opportunity of transferring new behaviours to other family members. More precisely, participants mentioned explaining the programme and its benefits to significant others. This ‘*knock on*’ effect was less common for exercise but was still described by some service users.

*“Yes, I also got my brother into it you know, my older brother he started coming to the gym with me. Plus when this is over my wife is coming to the gym with me.” (Service User 16)*

Socialising was often described as a challenging situation when trying to follow the lifestyle outlined in the programme.

*“When you go to somebody’s home and they’ve invited you in and they’ve prepared a meal for you, it’s very difficult to say, I won’t eat that. I can’t eat that. I shouldn’t eat that.” (Service User 3)*

##### Health-related outcomes

Participants also highlighted factors resulting from their participation in the NHS DPP. More precisely, how participating in the programme made them aware of how behaviour could affect their health, but also about how overall wellbeing was important.

*“I think that's important. It's not just diabetes. It's your overall, and you can't take one thing in isolation. You've got to have an overall overview and treat the whole person, basically.” (Service User 1)*

Service users also voiced disappointment when blood test results still identified them at high risk at the end of the programme. This feeling was increased when the programme ended for them even though they were still at risk.

*“Yes, towards the end of the programme I had a blood test and my blood sugar hadn’t gone down. (…) She was on the last one [session], so I’d had the blood sugar levels gone up the month before and then her last call and then that was it, it was, “Oh well, good bye, see you.” (…) There was no advice given, no further advice given, no, “Go on to such and such a thing.” I presume the thing was of course what I will do is go back and see my doctor. (…) I did lose 5% of my body weight, I am far fitter, I’m not objecting to it, it was a good wakeup call, it didn’t do me any harm. I still need to work now on the diabetes.” (Service User 19)*

##### Behavioural maintenance

Regular participation in the programme also exposed participants to content related to behavioural maintenance, particularly towards the end of the programme sessions. The importance of behaviour change maintenance was raised by deliverers.

*“For people to gain knowledge in order to maintain and sustain the behaviour changes that we are hopefully implementing throughout the programme. For them to carry on and live a longer, healthier life, I suppose, without that hand-holding support.” (Deliverer 9)*

The discontinuation of active support and maintenance of behavioural change outside of the programme was also raised as a challenge, for instance the costs associated with gym membership.

*“[Gym membership] I got 12 months, which finishes in July. I don’t know whether I’ll be able to afford it after that. I don’t know.” (Service User 15)*

#### Factors influencing the NHS DPP implementation

##### Case identification and referral

Referral was often described as challenging and complex in terms of identifying high risk patients from GP practices. The importance of the identification of eligible participants and the need to concentrate on that aspect was also highlighted. This linked to the suggestion of using community involvement so the workload was not solely on GP practices, but also to increase the reach of the programme.

*“Our community based approach to working in areas where we know there are large populations of at risk people. These are communities that don’t often engage with GPs and with other things, what they always call the hard to reach populations. They have been doing some very innovative work out there.” (Commissioner 3)*

Referrers highlighted the importance of the period between referral and starting the intervention and that delays could be a barrier to implementation of the programme.

*“I know that that organisation have realised that it’s unacceptable because there were cases where people could be waiting weeks to be contacted by the organisation. That’s a frustration and a challenge on our part because we’ve got these people; we’ve seen them in the community. It’s been a one-stop shop: we’ve got them assessed, we’ve done the blood test, they’ve turned out to be IGR; they’ve been really motivated. Then, because of red tape, then that person has been stuck in sort of a triage queue.” (Referrer 2)*

Referrers suggested the importance of having an in depth understanding of the lifestyle intervention in order to provide accurate information to users at this stage. The potential of providing brief advice at this stage was also mentioned by referrers. In line with this, specific training in diabetes prevention, behaviour change and motivational interviewing was viewed as crucial.

*“That’s obviously where our guys are skilled, because they know about all the links of lifestyles to the modifiable factors with diabetes etc. So, they can go, they can use motivational interviewing and really sit there and listen to that person, and then reflect anything back to them that they believe to be pertinent, and then get that person themselves to come up with a little bit of an action plan of what they’re going to do. As well as that, it’s all about getting them into the intervention (…).” (Referrer 2)*

Outreach strategies in specific communities were also suggested by different stakeholders (i.e. deliverers, referrers and commissioners) as an effort to get more people from BME communities referred to the programme.

*“We’ve got lots of links in [district] in [town] which has got a high Yemeni Muslim population, and we link in with the mosques there. We do our absolute best to make sure that we get into those communities. We link in with other organisations as well; there’s another sort of level: level zero engagement, social enterprises who’ve got specific BME teams. So, if we need people to translate or we need people who… If there’s a particularly hard-to-reach group who we’ve not got links with, we’ll use other agencies and work in partnership with them. They’ll help us access those groups and really sell the programme to them of why we should take part and why they should be screened for diabetes and impaired glucose regulation risk.” (Referrer 2)*

##### Delivery and content of the intervention

Deliverers suggested that scripts needed to allow for enough flexibility to adapt to specific needs (e.g. men-only sessions), any cultural sensitivities (e.g. healthy food for different ethnic groups, recipes in different languages) or any queries users might have. Some areas had a wide cultural diversity (e.g. language, ethnicity), different levels of disability or mental health problems with very different needs and expectations.

*“Culture wise, when we're talking about healthy food and eating, we need to bear in mind that people from one culture or religion might not drink alcohol or they might have - for example, we've got the Asians, they'll have the Asian diet and if we've got English people in the group, they'll have the English diet but it is fine. It works absolutely fine. Everybody knows about everybody and it does work out fine. We have relevant leaflets to hand out for those that might talk about the Asian foods or healthy tips on the Asian diet and then healthy tips on the English diet so that's fine. One thing that could be a barrier is your language but again, we're quite good like that because we've got quite a variety of champions. A lot of us are bilingual anyway.” (Deliverer 11)*

Interviewees also described that sessions were not long enough to deliver the specified content and provide one-to-one advice to patients that might need further support. Similarly, deliverers stated that group sizes needed to be manageable in order to provide personalised advice.

*“If it is small groups, that's fine. Obviously they get a bit more one to one which can be good as well. Sometimes if you've got a bigger group, time limit can be - you might go over a little bit of time just because you're filling out paperwork or you're having a bit of a session.” (Deliverer 11)*

##### Accountability/responsibility

Accountability and responsibility refers to the need to acknowledge who was responsible for what at different stages of the DPP pathway i.e. what identification strategies are commissioned within an area, who is responsible for identification and referral within each GP practice or who is responsible for how patient data is collected, recorded and shared.

Commissioners described the necessity to identify what pathways and referral routes are available and also try to identify which are working within their area.

*“It’s helped a lot in terms of refining pathways in terms of the evidence behind things, and what will and won’t work, but also in terms of; I think there’s an awful lot of information associated with the programme, and the expectations of commissioners, expectations of providers, and I think they’ve been slightly shifting as well as we’ve gone. Having conversations with people has helped clarify some of those issues, regarding at what point the pathway is the responsibility of commissioners, and at what point the pathway becomes the responsibility of the provider itself.” (Commissioner 5)*

A major challenge described by deliverers were the difficulties in getting referrals to the programme. These initial difficulties were related to organizational problems, such as having third party companies responsible for referral and different levels of engagement from GP practices.

*“Initially there were a few challenges at the beginning of the programme. The programme was supposed to roll out in early October but we didn’t really start to get referrals until December. So I think that some GPs were slow to take up the offer. I think, also, there was an assumption that GPs would buy into this. Actually some GP surgeries decided that they didn’t want to be part of the programme. We’ve had varying degrees of GP engagement.” (Deliverer 6)*

##### Fidelity

Fidelity procedures emerged in the form of service user satisfaction, outcome data collection and performing quality assurance assessments to monitor intervention delivery.

The tracking of patients throughout the process was raised as a complex but necessary component of the programme to ensure appropriate data collection systems and to make sure the programme is implemented as it should be. As well as tracking those who adhere to the programme the necessity to identity why patients did not take part or withdraw was raised to try and create solutions to this using targeted approaches.

*“Where now we've done it in a very systematic approach. We have a template on system one that's been developed just for phase one and phase two. So we're able to track the patient all the way through to understand what stage they went into the clinic, what stage if they were referred, when they were referred.” (Commissioner 2)*

Some sites had systems in place to collect feedback from participants about the programme. The feedback collected was used to support the development of case studies to show to current users or to use when advertising the programmes and improve service users’ engagement with the programme. Likewise, the creation of these successful case studies also denoted deliverers’ enthusiasm about and responsiveness to the NHS DPP.

*“We do collect a lot of qualitative feedback from the participants. Also, just if a member of staff has a patient who has made good progress or has made significant changes and has really been a success story, then we’ll do case studies on those people as well.” (Deliverer 1)*

Deliverers also mentioned the existence of annual assessment for staff and systems to monitor delivery and ensure it meets standard quality criteria. Nevertheless, deliverers reflected on the importance of having specific scripts about what to collect in terms of feedback and quality. Consistent and thorough measures of fidelity appeared to be lacking and required further consideration to better understand successful implementation.

*“So I think we need an audit of how well we’re doing now and bring together good practice so that all sessions have this quality assurance that they are as good as you can get.” (Deliverers 13)*

## Discussion

### Principal findings

In this qualitative study, we aimed to elicit stakeholders’ perceptions and experiences of participating in the intervention and implementing the NHS DPP during the demonstrator site phase. Our analysis identified factors that influenced participation and implementation of the NHS DPP, including: risk communication at referral; perceived impact of the NHS DPP on service users; maintenance of behavioural change; case identification procedures; adaptability of the intervention; responsibilities along the NHS DPP pathway; and fidelity of implementation.

Stakeholders identified the point of referral as a window of opportunity to offer brief advice and promote behaviour change, and to provide an understanding of the risk of T2D and intervention details. The provision of risk information during the NHS DPP referral process could take the form of a brief opportunistic intervention (i.e. an intervention that takes very little time). A number of systematic reviews have shown strong evidence that brief advice from physicians is effective for smoking cessation and some evidence that it is effective at reducing alcohol consumption [30–32]. Previous research supports the effectiveness of a behaviourally-informed, very brief, physician-delivered opportunistic intervention (i.e. 30 s intervention) for weight management [33].

Service users highlighted a range of factors linked to behaviour change as a consequence of the NHS DPP. These included understanding the potential of the intervention in preventing T2D; increased self-efficacy; and the ‘window of opportunity’ that changed their lives. They also drew attention to their need for support beyond the NHS DPP to help them maintain their changed behaviour, and associated costs. Theoretical explanations of behaviour change maintenance [34] emphasise the importance of factors including: maintenance motives, self-regulation, resources (psychological and physical), habits, and environmental and social influences.

In line with this, NHS DPP stakeholders described the importance of: maintenance motives to sustain behaviour change (e.g. blood test results at the end of intervention); adding maintenance resources as part of the NHS DPP so individuals can successfully maintain behaviour change; the environmental and social influences when transferring the new health behaviours to different contexts/settings. Despite voicing disappointment when blood test results still identified them at high risk at the end of the programme, service users highlighted overall satisfaction with weight loss outcomes that acted as motivation to maintain behaviour change. Evidence suggests that greater satisfaction with weight loss is predictive of maintained weight loss over time [35]. The importance of maintenance motives and environmental context and resources (e.g. cost of gym) have also been shown to be important for participants of a behavioural intervention to prevent T2D [24]. Maintaining change in physical activity and dietary behaviours requires an ongoing proactive effort and an intervention duration of more than 9 months might be needed [36].

Service users appreciated the peer support provided by being in a group, the positive social comparison, and the option of involving a significant person in the process. Service users also recognized their influence on others’ behaviour outside the sessions (‘*knock on*’ effects). There is evidence for the effectiveness of social support for behavioural change more generally [37] and also the particular importance of engaging support from a family member, friend or carer [38].

Intervention deliverers acknowledged the importance of following a structure or script for sessions but voiced the need to tailor their advice to service users’ social, cultural and individual preferences. Similarly, other studies have highlighted the importance of adapting health promotion interventions for ethnic minority communities and suggested that adaptation decisions should be based on detailed understanding of the target community [39–41].

In line with this, outreach initiatives in referral and recruitment and adaptations to the programme were viewed as essential to reach a wider at-risk population. Community engagement and outreach was identified by stakeholders as an important method of recruitment and awareness raising in addition to using GP practices and essential to target underrepresented groups (e.g. men, BME and deprived communities) and increase the reach of the NHS DPP programme. Stakeholders also highlighted the current pressures on primary care and the limited reach of letters sent by GPs as other reasons to implement more outreach strategies. Increased GP workload, concerns about lack of resources, and pessimism about the effectiveness of behavioural interventions has also been expressed by GPs as reservations about screening patients for impaired glucose tolerance [42]. Several strategies were implemented in the NHS DPP demonstrator phase, such as organising community-based screening events and to promote these within the ethnic minority communities, taking advantage of existing networks and religious groups and these strategies have also been found effective in previous literature [41]. Previous research also found the need to extend recruitment sources to the community and to community pharmacies in local areas [43].

Implementation fidelity is defined as the degree to which programmes are implemented as intended [44] and hence an important feature to be measured during implementation. Despite providing relevant information about various moderators of fidelity, including intervention complexity and participant responsiveness [45], our findings demonstrate the need for consistency in terms of how fidelity is measured across the different providers/locations. The findings also suggest that the measurement of fidelity within the NHS DPP could have been broader by including a measurement of different elements of adherence (i.e. content, coverage).

### Implications for practitioners and policymakers

The NHS DPP service specification could be improved by providing details on the use of effective risk communication techniques, whether verbal, written or both, and topics to be covered in the invitation to participate in the intervention. Providing a full explanation of the condition and their individual diagnosis was viewed as essential so that people understood why they have been referred and the benefits of attending the NHS DPP. Specific training on risk communication and in-depth knowledge about the intervention would be highly desirable for referrers. Introducing an overarching training component for both deliverers and referrers could improve fidelity and consistency of delivery [46].

The NHS DPP is offered to high-risk individuals during a minimum of 9 months and the final session involves signposting to local services that provide support to continue with improvements made to dietary and physical activity behaviours and weight loss. This session should also include strategies to support maintenance processes in behavioural interventions aimed at preventing T2D, such as providing resources to support successful behaviour change maintenance (e.g. discounted access to gym facilities), and reshaping the environment (e.g. introducing community-level support groups) [34, 47].

The need for more guidance about the group sessions’ content and the NHS DPP curriculum, whilst ensuring enough flexibility to address any cultural sensitivities or tailor to users’ preferences/needs was an important finding from the stakeholders’ interviews. Creating resources in different languages and content material aimed at different cultures/ethnicities (e.g. US DPP also available in Spanish [48]) could facilitate this [40, 49, 50].

### Strengths and limitations

A strength of the study was the inclusion of the full range of stakeholders (i.e. service users, intervention deliverers, referrers and commissioners) involved in the demonstrator phase of the NHS DPP, highlighting factors that were perceived as important for participation and implementation. This enabled stakeholder’s views to represent those involved at the start of commissioning a service right through to completion of the intervention and exit of the service. Interviews permitted insights into personal experiences at each of the seven demonstrator sites. The interviews were able to identify key findings in addition to recommendations and implications for policymakers and researchers. However, the scope of this work did not involve exploring any difference in the experiences across sites. The study was strengthened by using topic guides based on previous work in this area [36].

The service users were either completers of a NHS DPP intervention or were engaging with the NHS DPP sessions at the time of interview. Four withdrawers (i.e. people who did not complete the full programme) and one decliner (i.e. did not take up the offer to join the NHS DPP) were identified within one demonstrator site but no interviews were conducted due to inability to make contact. These interviews could have been important in identifying reasons why the intervention was not appealing or achievable for some individuals. Our interview attempts also illustrate the difficultly in gaining access to those who are not actively engaged in a behavioural intervention.

The lack of representation of ethnic minorities in this study is not representative of the population with diabetes and does not reflect the overall participation in the demonstrator phase. Barron and colleagues [51] showed attendance rates of 25% for Asian, Afro-Caribbean, mixed and other ethnic groups in the NHS DPP. These findings suggest that, in its development stage, the programme is reaching both those who are at greater risk of developing T2D and those who typically access healthcare less effectively. However, we do not know if the factors influencing participation and implementation described in this paper are representative of other ethnic groups. In addition, we are unable to determine whether non-English speakers were excluded from the list of contacts provided by the management team and therefore not represented in this study.

While the TDF provided a framework for some of the emerging themes (e.g. behavioural maintenance), most themes identified were better categorised outside the definitions of the theoretical domains (e.g. risk communication).

### Future research

Further investigation into withdrawers, decliners and service users from ethnic minorities could provide relevant information about the feasibility and acceptability of the programme and identify suggestions for further improvement. Future research could also examine retention levels and how outcomes are maintained in the long term. The latter would potentially be inexpensive and available as part of routine data collection implemented alongside the NHS DPP programme.

Research aimed at exploring the impact of providing structured and systematic brief advice as part of the NHS DPP could provide evidence to inform other potential changes to the NHS DPP service specification. This could be introduced by having a sub-sample of the NHS DPP implementing an enhanced risk communication package with process evaluation alongside.

Investigation into the different recruitment pathways implemented, such as GP practice identification or community outreach, would be informative to identify referral, participation and completion rates and how the different pathways compare. This could inform the NHS DPP service specification by providing further recommendations into different pathways for recruitment.

## Conclusions

The NHS DPP delivers a national evidence-informed behavioural intervention for prevention of T2D in England. Our findings highlighted factors that influenced participation in the intervention and the implementation of the NHS DPP. These factors included: risk communication at referral; perceived impact of the NHS DPP on service users; maintenance of behavioural change; case identification procedures; and adaptability of the intervention.

The NHS DPP will roll out to the whole country by 2020 with an expected 100,000 referrals available each year thereafter. This study provides evidence that could inform the further development of the NHS DPP service specification and improve the implementation of subsequent phases of the NHS DPP. Better risk communication and clearer specification would improve consistency at the referral stage and could benefit participation in the NHS DPP intervention. Cultural adaptations and outreach strategies could also ensure the NHS DPP contributes to reducing health inequalities.

## Supplementary information

**Additional file 1.** Summary of the NHS DPP demonstrator site interventions.

**Additional file 2.** Topic guides.

## Data Availability

The datasets used and/or analysed during the current study are available from the corresponding author on reasonable request.

## Abbreviations

NHS DPP: National Health Service Diabetes Prevention Program
T2D: Type 2 Diabetes
