# Supplementary material for: Stakeholders’ perceptions and experiences of the National Health Service diabetes prevention programme in England: qualitative study with service users, intervention providers and deliverers, commissioners and referrers
Source: BMC Health Serv Res. 2020 Apr 15;20:307. doi: 10.1186/s12913-020-05160-2 (PMC7158071; doi:10.1186/s12913-020-05160-2)
Supplement: Supplementary file 1 — Additional file 1. Summary of the NHS DPP demonstrator site interventions. [file 12913_2020_5160_MOESM1_ESM.docx]

Additional file 1

Summary about NHS DPP demonstrator site interventions

Components of the intervention

Both the national specification provided by Public Health England and NICE PH38 guidance clearly stated that the NHS DPP should encompass a behavioural intervention with three main goals: a) dietary improvements; 2) increased physical activity; and 3) weight reduction.

Most programmes developed by demonstrator sites included the content components specified in NICE PH38 guidance and the NHS DPP service specification: increasing physical activity was reported in six out of all seven sites; promoting weight loss in five out of all seven sites and improving dietary habits in six out of all seven sites.

The specific components of the intervention vary from site to site. Some demonstrator sites were using previously tested and implemented interventions (Site D, Site C, Site F). The lifestyle programme developed by Site D also included advice on stopping smoking. Site A mentioned the existence of a lifestyle management programme based on the Lighten Up service. Site B reported using a health trainers’ service, programme and a 12-week weight management programme. Site E had two interventions: 1) a tier 3 weight management programme and 2) a tier 2 weight management programme. In addition, the programme in Site E also included a wide range of physical activity interventions. Site F was implementing a structured education programme for diabetes, together with an exercise programme. Site G was implementing Walk Away from Diabetes (short course) and a nutritional component was under development.

Setting

The national service specification advised that this programme should be delivered in a range of groups within the community. NICE PH38 recommended delivery of programmes in a range of venues such as workplaces, leisure, community and faith centres, and outpatient departments and clinics. Most demonstrator site interventions were delivered in leisure and community centres (e.g. local authority facilities) or primary care premises (e.g. NHS facilities)

Format

Both the national service specification and NICE PH38 recommended that the interventions should be delivered in group sessions, face-to-face, but also provided the option for individual sessions to be included and to run at different times (i.e. evenings/weekends). NICE PH38 recommended that groups include 10-15 people.

The majority of demonstrator sites programmes were delivering interventions in a mixture of group sessions, face-to-face sessions, and individual sessions. An exception was the Site F programme as it involves a telephone-based intervention. Site C and Site G also included a digital component in their programmes.

None of the demonstrator sites relied solely on online provision of their intervention programmes. In light of this, NHS Choices online weight loss programme was identified and included in this mapping exercise, in order to provide an alternative type of intervention and shed some insight on digital interventions options. The NHS Choices programme features an online 12-week weight loss plan with several components. Individuals can access all materials on the NHS Choice website by self-referring themselves to the weight loss plan. An initial self-assessment of BMI is necessary and can be done by using the BMI calculator. Other features of this programme include a calorie checker, running podcasts (e.g. ‘couch to 5k’), other videos and a weight loss forum.

***Tailoring***

The national programme specification did not include information about tailoring of these interventions. NICE PH38 recommended that lifestyle-change programmes should provide tailored advice about diet, physical activity and included information about techniques to use to help change behaviour.

Some demonstrator site programmes seemed to provide personalised components of their intervention, mostly for physical activity. Nevertheless, some information regarding the extent and nature of tailoring was unclear in the provided documents and a formal request on this matter to demonstrator sites will be made to clarify this aspect.

***Type of session***

Both the national specification and NICE PH38 recommended referring people to an exercise referral scheme or supervised exercise sessions, as part of an intensive lifestyle-change programme.

The type of sessions offered by demonstrator sites were mostly described as weight loss consultations, exercise referrals/advice and sessions run by health trainers. Site E was implementing mostly counselling-based sessions. Sessions involving supervised physical activities were offered in five demonstrator sites. Sessions described as structured education were implemented in two sites.

***Intervention outcome targets***

Both the national specification and NICE PH38 recommended the following specific targets for participants: undertake a minimum of 150 minutes of ‘moderate-intensity’ physical activity per week; gradually lose weight to reach and maintain a BMI within the healthy range (with an initial target of 5-10% weight loss); increase their consumption of wholegrains, vegetables and other foods that are high in dietary fibre; reduce the total amount of fat in their diet; and eat less saturated fat.

Most interventions did not seem to specify intervention outcome targets for their programmes in terms of physical activity or diet. Only four demonstrator sites specified intervention targets for weight loss (i.e. at least 5% of initial body weight).

***Behaviour Change Techniques*** (please see Appendix E for definitions)

The national specification suggested including (but not limited to) goal setting and self-monitoring. NICE PH38 recommended using the following BCTs: information provision; motivational interviewing; goal setting; action planning; coping planning; relapse prevention; self-regulation techniques; and social support.

There was limited information about use of behaviour change techniques (BCTs) in baseline documents from demonstrator sites. Two sites mentioned using information provision, motivational interviewing, action planning, coping planning, relapse prevention, self-regulation techniques; goal setting; four sites described the use of goal setting and both motivational interviewing and action planning in their programme; and social support was reported in one site.

***Intensity and duration***

The national specification suggested that the programme should be delivered in a series of sessions, and include at least 13 sessions across 9 months (minimum duration). NICE PH38 suggested at least 8 sessions, with a minimum contact of 16 hours (1-2 hours per session), and weekly or fortnightly sessions that reduce in intensity over time. The duration of the programme should be at least 9-18 months.

The intensity of interventions differed greatly between sites. The intensity of sessions across the DPP programmes varied from six sessions to 52 sessions, with some sessions being held twice a week (Site C) and others monthly (Site F, Site G).

The duration of the NHS DPP programmes also varied greatly between demonstrator sites, with some being only six weeks (Site B) and other lasting up to 12 months (Site D). Only three demonstrator sites were compliant with the recommended duration of the NHS DPP programme.

**Table: Components of the NHS DPP intensive lifestyle change intervention detailed by demonstrator sites and NHS choices weight loss programme in comparison to component specification in NICE PH38 guidance and the NHS DPP National Service Model**

| **Intervention components** | | **NICE PH38** | **NHS DPP** | | **Site A** | | **Site B** | | **Site C** | | **Site D** | | **Site E** | | **Site F** | | **Site G** | |
| --- | --- | --- | --- | --- | --- | --- | --- | --- | --- | --- | --- | --- | --- | --- | --- | --- | --- | --- |
| **Components** |  | |  |  | |  | |  | |  | |  | |  | |  | |  |
| PA^a^ | ✓ | | ✓ | **?** | | ✓ | | ✓ | | ✓ | | ✓ | | ✓ | | ✓ | |  |
| Weight Management | ✓ | | ✓ | ✓ | | ✓ | | ✓ | | **?** | | ✓ | | **?** | | ✓ | |  |
| Diet | ✓ | | ✓ | **?** | | ✓ | | ✓ | | ✓ | | ✓ | | ✓ | | ✓ | |  |
| **Providers** |  | |  |  | |  | |  | |  | |  | |  | |  | |  |
| Primary healthcare professionals | ✓ | | ✓ | ✓ | | ✓? | | ✓? | | ✓ | | ✓? | | 🗶 | | 🗶 | |  |
| NHS specialist | ✓ | | ✓ | 🗶 | | 🗶 | | 🗶 | | 🗶 | | 🗶 | | ✓ | | 🗶 | |  |
| LA^b^ | ✓ | | ✓ | ✓ | | ✓ | | ✓ | |  | | ✓ | | ✓ | | 🗶 | |  |
| Other | ✓ | | ✓ | 🗶 | | 🗶 | | ✓ | | ✓? | | ✓ | | 🗶 | | ✓ | |  |
| **Setting** |  | |  |  | |  | |  | |  | |  | |  | |  | |  |
| Workplaces | ✓ | | 🗶 | 🗶 | | 🗶 | | 🗶 | | 🗶 | | 🗶 | | 🗶 | | 🗶 | |  |
| Leisure, community and faith centre | ✓ | | 🗶 | ✓? | | ✓? | | ✓? | | ✓? | | ✓ | | ✓? | | ✓ | |  |
| Health care | ✓ | | 🗶 | ✓ | | ✓ | | ✓ | | ✓ | | 🗶 | | 🗶 | | 🗶 | |  |
| Other? | ✓ | | 🗶 | 🗶 | | 🗶 | | 🗶 | | 🗶 | | 🗶 | | 🗶 | | 🗶 | |  |
| **Format** |  | |  |  | |  | |  | |  | |  | |  | |  | |  |
| Group 10-15 ppl | ✓ | | ✓ | 🗶 | | ✓ | | ✓ | | ✓ | | ✓ | | 🗶 | | ✓ | |  |
| Face-to-face | ✓ | | ✓ | ✓? | | ✓ | | ✓ | | ✓ | | ✓ | | ✓ | | ✓ | |  |
| Individual | 🗶 | | ✓ | ✓? | | ✓ | | ✓ | | ✓? | | 🗶 | | 🗶 | | 🗶 | |  |
| Digital | 🗶 | | 🗶 | 🗶 | | 🗶 | | ✓ | | 🗶 | | 🗶 | | ✓ | | ✓ | |  |
| **Tailoring** |  | | 🗶 | ✓? | | ✓ | | 🗶? | | ✓? | | ✓? | | ✓? | | 🗶? | |  |
| PA^a^ | ✓ | |  |  | |  | |  | |  | |  | |  | |  | |  |
| Diet | ✓ | |  |  | |  | |  | |  | |  | |  | |  | |  |
| Weight management | ✓ | |  |  | |  | |  | |  | |  | |  | |  | |  |
| **Type of sessions** |  | |  |  | |  | |  | |  | |  | |  | |  | |  |
| Physical activity | ✓ | | ? | 🗶 | | ✓ | | ✓ | | 🗶 | | ✓ | | ✓ | | ✓ | |  |
| Counselling | ✓ | | ? | ✓? | | ✓ | | ✓ | | ✓ | | ✓ | | ✓ | | 🗶 | |  |
| Educational | 🗶 | | ? | 🗶 | | 🗶 | | 🗶 | | 🗶 | | 🗶 | | ✓? | | ✓ | |  |
| Digital/remote | 🗶 | | 🗶 | 🗶 | | 🗶 | | 🗶 | | 🗶 | | 🗶 | | ✓ | | 🗶 | |  |
| **Targets** |  | |  | 🗶 | | 🗶 | |  | | 🗶 | |  | |  | | 🗶 | |  |
| PA^a^ | ✓ | | ✓ |  | |  | | ? | |  | | ? | | ? | |  | |  |
| Diet | ✓ | | ✓ |  | |  | | ? | |  | | ? | | ? | |  | |  |
| Weight management | ✓ | | ✓ |  | |  | | ✓ | |  | | ✓ | | ✓ | |  | |  |
| **BCTs^c^** |  | |  |  | |  | |  | |  | |  | |  | |  | |  |
| Information provision | ✓ | | ? | ? | | ? | | ? | | 🗶 | | 🗶 | | 🗶 | | ? | |  |
| Motivational interviewing | ✓ | | ? | ? | | ? | | ? | | 🗶 | | 🗶 | | ✓ | | ? | |  |
| Goal setting | ✓ | | ✓ | ? | | ? | | ? | | ✓ | | ✓ | |  | | ? | |  |
| Action planning | ✓ | | ? | ? | | ? | | ? | | 🗶 | | 🗶 | | ✓ | | ? | |  |
| Coping planning | ✓ | | ? | ? | | ? | | ? | | 🗶 | | 🗶 | | 🗶 | | ? | |  |
| relapse prevention | ✓ | | ? | ? | | ? | | ? | | 🗶 | | 🗶 | | 🗶 | | ? | |  |
| Self-regulation techniques | ✓ | | ✓ | ? | | ? | | ? | | 🗶 | | 🗶 | | 🗶 | | ? | |  |
| Social support | ✓ | | ? | ? | | ? | | ? | | 🗶 | | 🗶 | | 🗶 | | ? | |  |
| **Duration** |  | |  |  | |  | |  | |  | |  | |  | |  | |  |
| min 9-18 months | ✓ | | ✓ | ? | | ✓ | | 🗶 | | 🗶 | | ✓ | | 🗶 | | 🗶 | |  |
| Follow up every 3 months for 2 years | ✓ | | ✓ | ? | | 🗶 | | ✓ | | 🗶 | | 🗶 | | 🗶 | | 🗶 | |  |
| **Intensity** |  | |  |  | |  | |  | |  | |  | |  | |  | |  |
| Min. 8 sessions (min. 16h) | ✓ | | ✓ | ? | | ✓ | | ✓ | | ? | | ✓ | | 🗶 | | 🗶 | |  |
| Weekly or fortnightly | ✓ | | 🗶 | ? | | ✓ | | ✓ | | ? | | ? | | ✓ | | 🗶 | |  |

✓ Done; 🗶 Not done; **?** Unclear

^a^PA – Physical activity; ^b^LA - Local Authority ; ^c^BCTs – Behaviour Change Techniques
