# Supplementary material for: Stakeholders’ perceptions and experiences of the National Health Service diabetes prevention programme in England: qualitative study with service users, intervention providers and deliverers, commissioners and referrers
Source: BMC Health Serv Res. 2020 Apr 15;20:307. doi: 10.1186/s12913-020-05160-2 (PMC7158071; doi:10.1186/s12913-020-05160-2)
Supplement: Supplementary file 2 — Additional file 2. Topic guides. [file 12913_2020_5160_MOESM2_ESM.docx]

**Additional file 2: Topic guides**

**NHS DPP Commissioner Interview topic guide (IRAS number 190418)**

Thank you for agreeing to take part in this interview. We are inviting a number of commissioners so I am pleased you are able to come along and help. We are interested in your behaviour (in commissioning the NHS DPP (programme)), your perception of the impact on your clients and ways to help more people in the future.

| 1. **COMMISSSIONING THE PROGRAMME:** |
| --- |
| **Intentions and goals** |
| Why interested in commissioning the NHS DPP (programme) (health, community, making a difference)?  What do you hope to achieve by commissioning the NHS DPP (programme)? |
| **Reinforcement** |
| What feedback do you receive from the NHS DPP (programme)? |
| **Knowledge** |
| What knowledge do you need in order to decide whether to commission this programme?  Are there any benefits of the NHS DPP (programme) over similar interventions you commission?  Are there any disadvantages of the NHS DPP (programme) over similar interventions you commission? |
| **Nature of the behaviour** |
| Outline the programme that has been commissioned  How does commissioning the NHS DPP differ from other programmes? |
| **Social influences on you as a commissioner** |
| What social and cultural facilitators and barriers are there to consider in commissioning this programme?  Have you spoke with other commissioners about the NHS DPP (programme)? If so what did you learn from speaking to others? |
| **Beliefs about capabilities and skills** |
| How confident are you in the skills and capability of the deliverers/providers to deliver this programme?  Is there any training that would be needed?  How would you organise staff training for the NHS DPP (programme)?  What are the challenges in your work? |
| **Behavioural regulation, attention and decision processes** |
| How will you decide whether to commission the NHS DPP (programme) in the future? |
| **Environmental context and resources** |
| What staff, facilities and resources are needed in enable to implement the NHS DPP (programme)? |
| **Social role and identity** |
| What is your remit as commissioner of the NHS DPP (programme)? (your role / what you do)  Do you have competing roles? |
| **Emotion** |
| How important is diabetes prevention in general? |
| ANYTHING ELSE ABOUT COMMISSIONING THE PROGRAMME (YOUR OWN BEHAVIOUR) |

| 1. **INTERVENTION IMPACT: your perception of your clients / programme participants** |
| --- |
| **Intentions and goals** |
| What do you think are the important barriers and facilitators for people joining the NHS DPP (programme)? |
| **Reinforcement** |
| What do you think helps people to stay motivated? |
| **Knowledge** |
|  |
| **Nature of the behaviour** |
|  |
| **Social influences on your clients** |
| How is your commissioning affected by social and cultural factors of people you wish to engage with the NHS DPP (programme)? |
| **Beliefs about capabilities and skills** |
| Do you think the information provided in each session is adequate? |
| **Behavioural regulation, attention and decision processes** |
| How do you know the intervention is delivered as planned/commissioned? |
| **Environmental context and resources** |
| Where does the intervention take place?  Are there any problems with finding suitable venues?  What are the issues around access to the NHS DPP (programme) (literacy, finance, culture, venue)? |
| **Social role and identity** |
|  |
| **Emotion** |
|  |

**NHS DPP Health care professional Interview topic guide (IRAS number 190418)**

Thank you for agreeing to take part in this interview. We are inviting a number of health care professionals so I am pleased you are able to come along and help. We are interested in your behaviour (in referrals to the programme), your perception of the impact on your clients and ways to help more people in the future.

| 1. **REFERRALS TO THE PROGRAMME:** |
| --- |
| **Intentions and goals** |
| What motivated you to refer patients to the NHS DPP (programme) (health, community, making a difference)?  What do you hope to achieve by referring patients to the NHS DPP (programme)? |
| **Reinforcement** |
| What feedback do you receive from the NHS DPP (programme)?  Do you receive any feedback from patients after referral to the the NHS DPP (programme)?  How do you find out the effect of the NHS DPP (programme) on your patient? |
| **Knowledge** |
| How did you find out about the NHS DPP (programme)?  How is it promoted in your area? |
| **Nature of the behaviour** |
| What is the best way to refer patients to the NHS DPP (programme)?  What is the best way to communicate risk of diabetes to your patients? |
| **Social influences on you as a health professional** |
| What social and cultural facilitators and barriers are there to referring patients to this programme?  Have you spoke to other health care professionals about the DPP? If so what did you learn from speaking to others? |
| **Beliefs about capabilities and skills** |
| How confident are you in your ability to refer patients to the NHS DPP (programme)?  How confident are you that the providers have the skills and capability to deliver this programme?  Do you or your staff need any training about how to refer patients to the NHS DPP (programme)?  What are the challenges in referring patients to the NHS DPP (programme)? |
| **Behavioural regulation, attention and decision processes** |
| How do you decide whether to refer to this programme?  How do you assess whether a patient is eligible? |
| **Environmental context and resources** |
| How has referring to the NHS DPP (programme) impacted on your surgery? |
| **Social role and identity** |
| What is your remit as referrer to the NHS DPP (programme)? (your role / what you do) |
| **Emotion** |
| How important is diabetes prevention in general? |
| ANYTHING ELSE ABOUT REFERRING TO THE PROGRAMME (YOUR OWN BEHAVIOUR) |

| 1. **INTERVENTION IMPACT: your perception of your clients / programme participants** |
| --- |
| **Intentions and goals** |
| What do you think influences patients to join the NHS DPP (programme)?  What do you think are the important barriers and facilitators for patients joining the NHS DPP (programme)? |
| **Reinforcement** |
| What do you think helps patients to stay motivated? |
| **Knowledge** |
| What do you need to know about the information provided to patients within the NHS DPP (programme)?  How could this knowledge be best presented/provided to you? |
| **Nature of the behaviour** |
|  |
| **Social influences on your clients** |
| How is you referral affected by social and cultural factors of patients you wish to engage in the NHS DPP (programme)? |
| **Beliefs about capabilities and skills** |
| Do you think the information provided in each session is adequate? |
| **Behavioural regulation, attention and decision processes** |
| How do you know that the interventions are delivered as planned/commissioned? |
| **Environmental context and resources** |
| Where does the intervention take place?  What are the issues around access to the NHS DPP (programme) (literacy, finance, culture, venue)? |
| **Social role and identity** |
|  |
| **Emotion** |
|  |

**NHS DPP Programme deliverer/provider topic guide (IRAS number 190418)**

Thank you for agreeing to take part in this interview. We are inviting a number of programme deliverers/providers so I am pleased you are able to come along and help. We are interested in your behaviour (in delivering the programme), your perception of the impact on your clients and ways to help more people in the future.

| 1. **DELIVERING THE PROGRAMME:** |
| --- |
| **Intentions and goals** |
| Why interested in delivering the programme, what motivates you (relatives, and friends, community, making a difference)?  What do you hope to achieve by delivering the programme? |
| **Reinforcement** |
| How rewarding is delivering this programme? Do you receive any feedback?  What is the best thing about your job?  How could the programme be improved? |
| **Knowledge** |
| What do you need to know to deliver this programme?  What training did you receive?  Was the training you received enough?  Do you need refresher training?  Did the training include watching other providers? If so what did you learn? |
| **Nature of the behaviour** |
| Outline one session you have delivered  What did it feel like to deliver this session? Was it as expected? Did you practice / repeat? |
| **Social influences on you as a trainer** |
| What social and cultural factors are there in delivering this programme? (time of sessions, group gender) |
| **Beliefs about capabilities and skills** |
| How confident are you in your skill and capability to deliver this programme?  Are you more confident in some areas (activities) than others?  What are the challenges in your work? |
| **Behavioural regulation, attention and decision processes** |
| How do you decide what to do in each session? Do you remember everything you should / could show people?  How do you prepare for each session? |
| **Environmental context and resources** |
| How convenient is the venue for you? Anything else important about this venue for you?  What equipment is available/needed for the classes? |
| **Social role and identity** |
| What do you do as a deliverer of the NHS DPP / programme? |
| **Emotion** |
| How important do you think diabetes prevention is in general? |

**NHS DPP Service user interview topic guide (IRAS number 190418)**

Thank you for agreeing to take part in this interview. We are inviting service users so I am pleased you are able to come along and help. We are interested in your feedback on the diabetes prevention programme.

*Black – Participators in the DPP

*Blue – Participators that withdrew from the DPP

* Red – Eligible patients that did not go on to take part in the DPP

* Purple – Patients not offered participation in the DPP

| **What can you tell me about the Diabetes Prevention Programme?** |
| --- |
| **Intentions and goals** |
| How did you find out about the programme?  Why did you become involved with the programme (GP referral, aware of a need for more support, relatives, and friends, community)?  Did timing have an effect on joining the programme? (retirement, new job, readiness to change)  Same 3 questions as above  What affected your intentions to be part of the DPP?  Have you heard of the NHS DPP?  Why did you choose not to join the programme when you were found to be eligible to take part? What influenced this decision?  Have you heard of the NHS DPP?  Would you have been interested in taking part in a DPP? What would influence your decision (doctor, others, word of mouth, timing)? |
| **Reinforcement** |
| What facilitated your continued involvement in the project? (personal experience of a friend or family, convenience, incentives)  What were barriers? (time, cost)  What factors lead to you stopping your participation in the DPP?  What barriers stopped you from taking part in the programme?  What would facilitate your involvement? Or act as barriers? |
| **Knowledge** |
| What do you know about diabetes prevention?  What did you expect/wish from a diabetes prevention programme?  What could be added to the programme?  Same questions as above  What do you know about diabetes prevention? Did you want to gain more information? What would you expect/wish from a diabetes prevention programme?  What do you know about diabetes prevention? Would you want to gain more information? What would you expect/wish from a diabetes prevention programme? |
| **Nature of the behaviour** |
| What does the programme offer?  Describe one session you attended in the diabetes prevention programme.  What difference has the programme made to you? (weight/diet/physical activity)  Same questions as above  What led to your withdrawal from the programme?  How did your behaviour change after finding out you were eligible even though you didn’t take part in the DPP?  How would attending a DPP change your behaviour? (healthy eating, increase activity levels?)  What might have changed your mind in order for you to join the programme? |
| **Social influences on you as a DPP service user** |
| Are there any social and cultural barriers to you attending the programme?  Are there any solutions to the barriers?  How supportive are friends and family?  How supportive is the group you are in? How supportive is the deliverer?  Do you learn from other people in your group?  Same questions as those above. What social or cultural barriers led you to withdraw?  What social or cultural barriers that stopped you joining the DPP? Are there any solutions to the barriers?  Are there any social and cultural barriers that would stop attending the programme? Are there any solutions to the barriers? |
| **Beliefs about capabilities and skills** |
| How confident are you in doing physical activity?  How confident are you in managing your weight?  How confident are you in healthy eating?  Same questions as above. How well are you able to manage your health independently?  Same as first 3 questions. How well are you able to manage your health independently? (Without assistance?)  Same as first 3 questions. How well are you able to manage your health independently? (Without assistance?) |
| **Behavioural regulation, attention and decision processes** |
| How do you decide whether to attend each of the sessions in the DPP? (what factors into the decision)  Do you remember all the information that is provided to you during the sessions?  Why did you attend each of the sessions you did? How well do you remember information provided to you from the sessions you attended?  What made you decide that the DPP was something you didn’t want to do? Did you receive enough information to help you make this decision?  How would you make the decision to join the DPP? How would you decide whether to attend each of the sessions in the DPP? (What would factor into the decision?) |
| **Environmental context and resources** |
| How convenient is it for you to attend the sessions? (ease of access to venue / time/ frequency of sessions).  How well are necessary resources readily available to make and maintain lifestyle changes? (access to shopping facilities, exercise, time to pursue healthy activities)  How did you find the resources or information suggested/provided for you? (Any consideration of/issues with education levels, financial issues, gender issues or cultural beliefs?)  Same questions as above (phrase in past tense rather than present)  Would the programme have fit into your daily life? How convenient would it have been to attend the DPP?  Would the programme have fit into your daily life? How convenient would it have been to attend the DPP? |
| **Social role and identity** |
| What impact did being told you were at risk of developing diabetes have on you?  Same as above  Same as attenders  Have you ever been told you were at risk of developing diabetes? How did that make you feel? |
| **Emotion** |
| What do you feel like after attending the DPP? (Pleased, Feel-good)  How do you feel after withdrawing from the DPP? (Regret, Right decision, Unsure)  How do you feel about not attending the DPP? (Regret, Right decision, Unsure)  How do you feel about not being invited on the DPP? |
